# Oral Supplementation with Ultramicronized Palmitoylethanolamide for Joint Disease and Lameness Management in Four Jumping Horses: A Case Report

**DOI:** 10.3390/ani10091469

**Published:** 2020-08-21

**Authors:** Enrico Gugliandolo, Alfio Barbagallo, Alessio Filippo Peritore, Salvatore Cuzzocrea, Rosalia Crupi

**Affiliations:** 1 Department of Chemical, Biological, Pharmaceutical and Environmental Sciences, University of Messina, 98166 Messina, Italy; egugliandolo@unime.it (E.G.); aperitore@unime.it (A.F.P.); rcrupi@unime.it (R.C.); 2 Department of Veterinary Science, University of Messina, 98168 Messina, Italy; horsedoctor@libero.it; 3 School of Medicine, Saint Louis University, St Louis, MO 63104, USA

**Keywords:** navicular syndrome, lameness, hoof pain, lmitoylethanolamide palmitoylethanolamide, ALIAmides

## Abstract

**Simple Summary:**

This paper reports the efficacy of Ultramicronized Palmitoylethanolamide (PEA-um) supplementation for four show-jumping horses with lameness and joint disease. Joint disease is often associated with inflammatory states and pain that lead to lameness or impairment in athletic performances. PEA-um is a nutraceutical compound that is well-known for its anti-inflammatory and analgesic proprieties, and is widely used in human medicine and small animal veterinary medicine. Although it includes a small number of cases, our study describes for the first time the efficacy of the use of PEA-um in horses. PEA-um was introduced to the normal diet of four horses with non-responsive lameness and significant impairment of athletic performance. After four months of PEA-um supplementation, all horses showed remissions of lameness that led to their reintroduction into showjumping competitions without disease recurrence. Therefore, despite the small number of cases included in this study, the observations suggest that PEA-um may be beneficial in the maintenance of joint disease in athletic horses.

**Abstract:**

**Background:** Four show jumping horses were evaluated for non-responsive lameness, which caused their withdrawal from show jumping competitions. The clinical evaluation was performed by radiographic examination, flexion tests, diagnostic anesthesia and lameness evaluation using the American Association of Equine Practitioners (AAEP) scale. The diagnoses were a case of navicular syndrome, a complicated case of chronic navicular syndrome and arthrosis of the distal interphalangeal joint of the right anterior limb and two cases of distal intertarsal joint arthritis. Nutraceuticals are often an important management strategy or coadjutant of pharmacological therapies in joint disease. Ultramicronized Palmitoylethanolamide (PEA-um) is an endogenous fatty acid amide that is well-known for its anti-inflammatory and analgesic proprieties widely used in human medicine and small animal veterinary medicine. Although it includes a small number of cases, our study describes for the first time the efficacy of the use of PEA-um in horses. The four horses with non-responsive lameness and significant impairment in athletic performance were daily treated with PEA-um into their normal diet. After four months of PEA-um supplementation, all horses showed remissions of lameness that led to their reintroduction into showjumping competitions without disease recurrence. Therefore, despite the small number of cases included in this study, these observations suggest a good prospective for developing a controlled experiment to test PEA in a larger cohort of horses.

## 1. Introduction

Nutraceuticals are often used in the management of joint disease of athletic horses [1]. Lameness in horses is often associated with joint disease, pain; arthrosis and navicular syndrome are one of the most commonly encountered causes of lameness in many types of athletic horses, including show jumpers and dressage horses [2]. Lameness is an important limitation of the utility performance of horses, but above all, of the wellbeing of horses. Navicular syndrome has long been thought of as a single disease process; however, it is actually caused by the alteration of several closely related structures, including collateral ligaments of the navicular bone, the distal sesamoid ligament impar, the navicular bursa, the digital deep flexor tendon, and the navicular bone itself [3]. Alterations in the joint capsule often compromise the delicate joint components that can also affect all of the adjacent structures, causing an inflammatory state and thus resulting in a condition of pain sensitization, which is a cause of disability for both the sports performance and the well-being of horses [4]. While nonsteroidal anti-inflammatory drugs (NSAIDs) and corticosteroids remain important therapeutic resources for the treatment of overt clinical lameness, nutraceuticals are becoming increasingly used as a therapeutic and prophylactic treatment to minimize the side effects associated with prolonged NSAID and corticosteroid [1]. Often, joint diseases are associated with persistent inflammation and pain conditions. Treatment of these pathological conditions often requires a long-term pharmacological therapy. Therefore, a prolonged treatment or maintenance period of months or even years can be obtained with the use of drugs with a high safety profile [5]. The fatty acid amide Palmitoylethanolamide (PEA), with the chemical structure (2-hydroxyethyl) esadecanamide, is naturally present in animals and plants and is a natural endogenous mediator with a “pro-homeostatic” role, produced and released in response to different stimuli, from different cell types and tissues. Although there is growing interest in studying the physiological role of PEA, its role as a key mediator in endogenous protection mechanisms, particularly the endogenous mechanisms responsible for controlling immune and inflammatory responses, is widely recognized [6]. In fact, PEA produced on demand following harmful stimuli exerts an anti-inflammatory and pro resolving action in the control of different physiological processes, such as the activation of immune cells and inflammatory responses. Therefore, as demonstrated by some previous studies on humans and dogs, the use of endocannabinoid-related PEA in different pathologies is a useful anti-inflammatory and analgesic therapeutic strategy [7,8]. Because of its nature as an endogenous compound, it offers the advantage of being safe to use, avoiding side effects, and a “multi-modal” pharmacological action that cannot be reached with the drugs currently available. There is numerous experimental and clinical evidence supporting the efficacy of pharmacological treatment with PEA in both human and veterinary medicine, ranging from the treatment of inflammatory pathology, skin disorders, and osteoarticular pathologies to pain treatment [9,10,11].

## 2. Materials and Methods

### 2.1. History and Case Presentation

All horses were residents of the same jumping facility located in Messina, Italy (body weight 450 ± 35 kg) and were housed in individual box stalls (3.50 × 3.50 m) under a natural photoperiod and indoor temperature. They were trained by the same professional rider. The training arena consisted of silica sand mixed with non-woven fabric, with a depth of 7 cm regularly maintained. Until the onset of lameness, the horses were trained regularly by the same trainer 6 days a week, with a day of rest, in particular 5 min of walking, 15 min of trotting, and 10 min of cantering, with one jumping session per week; 400 m length with 12 obstacles, including 7 vertical obstacles and 5 large obstacles of 1.10 ± 0.10 m in height. The horses participated in show jumping competitions once a month (2/3 day events, class 120/130 height). No ethical review was required as this is a case report. All the owners of the horses described in the present case gave their consent for publication.

**Figure 1:**
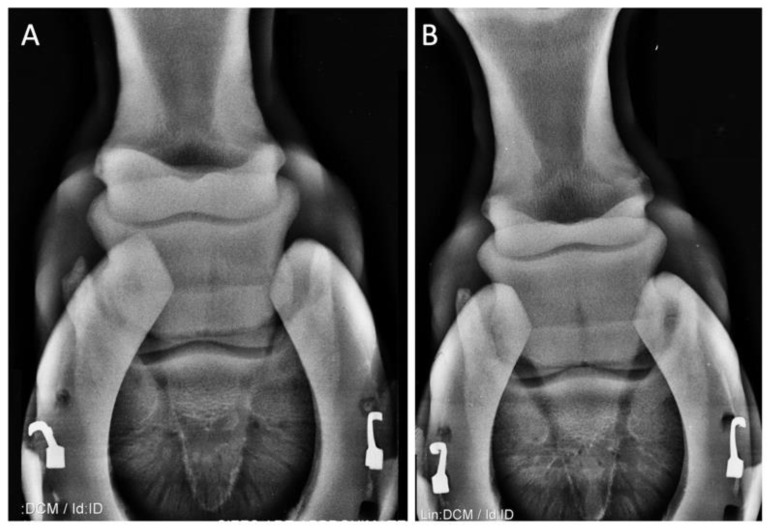
Case 1: A gelding 7-year-old jumping horse. X-ray of left (**A**) and right (**B**) forelimb. Panel A shows slight alterations in the silhouette of the navicular bone.

Case 1: A 7-year-old gelding jumping horse born in Italy. Presented for an investigation of left forelimb lameness of an approximately 1-month duration, with no obvious history of trauma. Conservative rest therapy and treatment with phenylbutazone for 5 days, corrective shoeing, and a gradual return to exercise over a 4-week period did not improve lameness. Lameness evaluation showed an inconsistent grade I out of V on AAEP scale lameness on the left forelimb, and the lameness was more obvious when lunged with the lame leg on the outside of the circle and on a hard surface. Based on radiographic (Figure 1) and clinical evaluation, the horse was diagnosed with navicular syndrome.

**Figure 2:**
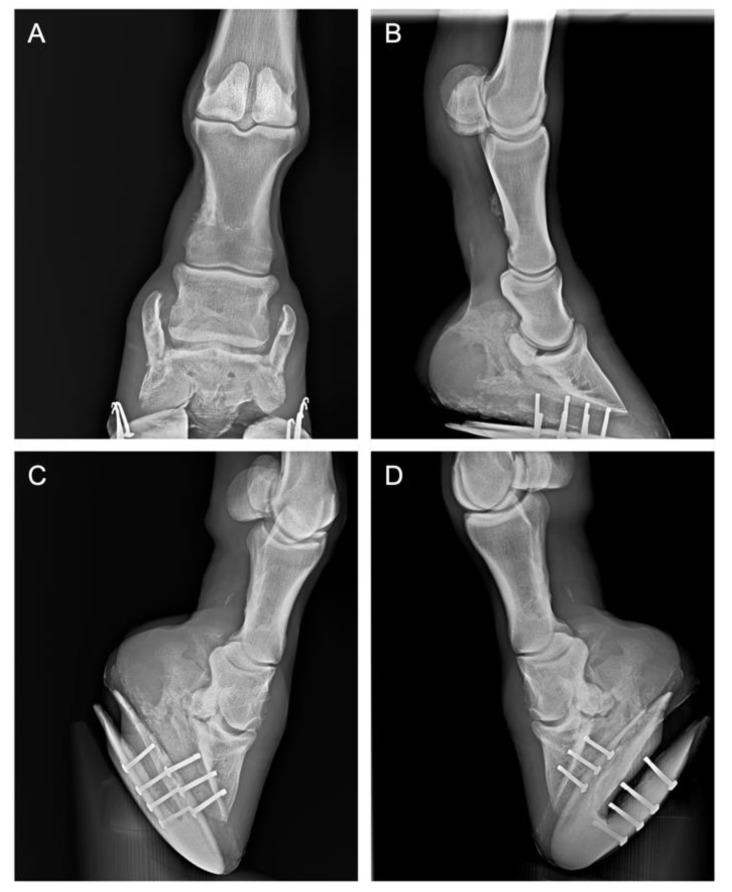
Case 2: X-ray of the right forelimb lateromedial, dorso-palmar, dorsoproximal–plantarodistal oblique, and plantaroproximal-plantarodistal oblique views (**A**–**D**) respectively revealed bone remodeling of the distal interphalangeal joint of the affected limb, periarticular osteophytes, and ossification of the collateral cartilage (sidebone), accompanied by alterations in the silhouette of the navicular bone.

Case 2: A 16-year-old Nederlands Rijpaarden en Pony Stamboek (NRPS) gelding jumping horse, presented for an investigation of right forelimb lameness of an approximately 1-month duration, with no obvious history of trauma. Conservative rest therapy and treatment with phenylbutazone or glucocorticoids and a gradual return to exercise over a 4-week period did not improve lameness. Lameness examination showed a consistent lameness of grade III out of V on the AAEP scale on the right fore limb at the straight trot and on the lunge in both directions. On both a hard surface and soft surface, the lameness was exacerbated by the limb flexion test. The lameness was more obvious when lunged with the lame leg on the outside of the circle. As shown in Figure 2, radiographic examination of the foot, including lateromedial, dorso-palmar, dorsoproximal–plantarodistal oblique, and plantaroproximal–plantarodistal oblique views, revealed important bone remodeling of the distal interphalangeal joint of the affected limb, periarticular osteophytes, and ossification of the collateral cartilage (sidebone), accompanied by alterations in the silhouette of the navicular bone. Based on these findings, the horse was diagnosed with chronic navicular syndrome and distal interphalangeal joint arthrosis of the right anterior limb.

**Figure 3:**
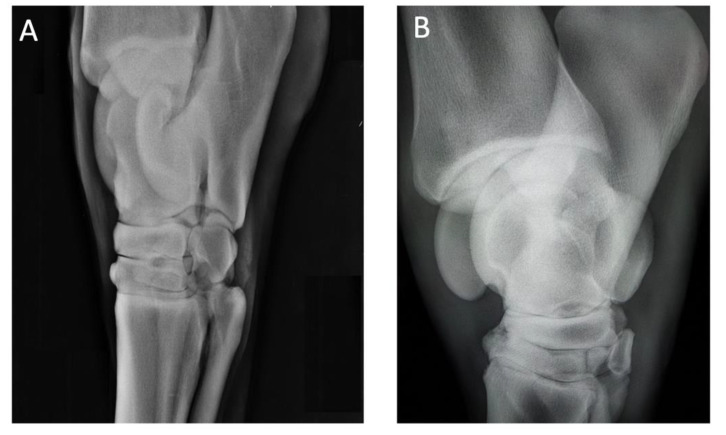
X-ray of the right hindlimb lateromedial. Panel (**A**): Case 3, a 14-year-old Holstein mare jumping horse. Panel (**B**): Case 4, a 15-year-old NRPS mare.

Case 3 and 4: A 14-year-old Holstein mare jumping horse with consistent grade II out of V on AAEP scale lameness and a diagnosis of distal intertarsal joint arthritis, as shown in Figure 3A. Additionally, a 15-year-old jumping NRPS mare presented consistent grade III out of V on AAEP scale hind limb lameness, and diagnosis of distal intertarsal joint arthritis, as shown in Figure 3B.

### 2.2. Clinical Evaluations

A thorough examination of lameness was conducted, which included detailed palpation of the extremities and back including overall inspection of the horses from all angles regarding general body condition, posture and weight bearing on the limbs, skeletal and soft tissue symmetry, localization of swellings and a detailed evaluation of the affected limb and its joints (inspection, palpation, manipulation) to reveal deformity, skin wounds, muscle wasting and to detect heat, pain, swelling, thickening, crepitation, restricted movement or instabilities. To better identify asymmetries, the investigation of the contralateral limb was carried out as well. Clinical tests were performed in all horses by two professionally licensed veterinarians (F.I.S.E. and FEI accredited) with more than 20 years of experience in the field, and one of the authors, A.B., who clinically followed the horses and supervised the clinicians. Although not completely blinded to the treatment, the two clinicians were unaware of the horse’s name or treatment, before examining and recording their lameness scores. This may increase the validity of the results, even if it does not completely address the reduction of the bias to a minimum and does not solve the problem of subjective variability in the evaluation of lameness and AAEP scoring. Diagnostic anesthesia with mepivacaine 2% was performed using the standard aseptic technique as previously described [12]. The lameness was considered localized to a specific joint when the subjective lameness grading decreased by at least 1 grade (AAEP score) within 10 min. Using routine settings, standard digital radiographic projections were acquired for those joints that responded positively to diagnostic anesthesia. In addition, the responses of the horses to the flexion test were recorded. The flexion test was done by keeping the joint of the affected limb in a strongly flexed position for 1 min. Subsequently, the limb was released, and the horse was observed by the veterinarian during the immediate trotting movement. The horse was trotted in a straight line for at least 20–30 m, in both directions [13]. The results were on a scale of 0 to 3 [14] (0: no reaction/negative; 1: mild reaction; 2: moderate reaction; 3: severe reaction). Follow up included re-evaluation of lameness and response to flexion test of the horse and the exact follow-up time was recorded for each case. Lameness during all observational periods was evaluated according to AAEP [15]. The scale ranges from zero to five, with zero being no perceptible lameness, and five being most extreme.

### 2.3. PEA-un Supplementation

Utramicronized Palmitoylethanolamide (PEA-um) Alilamid^®^ was provided by Innovet italia s.r.l. (saccolongo, PD, Italy). Horses were regularly fed with PEA-um (2.5 g) mixed with a regular mixture of cereals every day in the last ration of the day (19:00 p.m.). No supplement residues were observed in the manger. Horses were regularly fed three times a day (7:00 a.m., 13:00 p.m., and 19:00 p.m.) with standard ration comprised of hay (first cut meadow hay, sun cured, late cut, 8 kg/horse/day, 6.9% crude protein on average) and a commercial mixture of cereals: oat barley corn, about 3.5 kg/day: crude protein 14.5%, oil and fat 3.5%, crude cellulose 11%, carbohydrates NSC 36%, carbohydrates SC 24% (Destriero equimix Galtieri, Modugno, BA, Italy). Water was available ad libitum.

## 3. Results

### Effect of PEA-um Supplementation and Lameness Management

Table 1 shows the timeline since the start of treatment with PEA-um for Case 1, a 7-year-old gelding jumping horse with a lameness of grade I out of V on the AAEP scale due to a diagnosis of navicular syndrome. Table 1 (2) shows the clinical outcomes for case 2, a 16-year-old gelding jumping horse with lameness of grade III out of V on the AAEP scale on the right fore limb due to chronic navicular syndrome and distal interphalangeal joint arthrosis of the right anterior limb. One month after PEA-um treatments, the horses showed a significative improvement in lameness; however, only for case 2, an inconsistent grade II out of V was observed. The lameness was more obvious when lunged with the lame leg on the outside of the circle.

Lameness was exacerbated by proximal limb flexion, but not distal limb flexion. Subsequently, the horses were trained for the first week with 20 min of walking, and then with 20 min of walking, 5 min of trotting, and 5 min of cantering. Clinical observation after a period of 3 months from the start of the PEA-um treatment, showed a complete remission for lameness in case 1, while for case 2, the horse showed an inconsistent grade I out of V lameness. Lameness examination showed no lameness on the right fore limb at the straight trot and no lameness on the lunge in both directions. The lameness was more obvious when lunged with the lame leg on the outside of the circle. Lameness was slightly exacerbated by proximal limb flexion, but not distal limb flexion. In conclusion, after 3 months from the treatment with PEA-um and, for case 2, after a period of four months, the horse returned to complete training and competitions 6 days a week with a day of rest (5 min of walking, 15 min of trotting, and 10 min of cantering, with one jumping session per week; 400 m length with 12 obstacles, including 7 vertical obstacles and 5 large obstacles of 1.10 ± 0.10 m in height) and participating in show jumping competitions once a month (class 120/130 height) and, to date, there have been no recurrences in lameness.

The timelines of clinical outcomes for case 3 and case 4 were shown in Table 1. In particular, grade II out of V and grade III out of V on AAEP scale hind limb lameness were recorded as baseline for cases 3 and 4, respectively. Both cases had a diagnosis of distal intertarsal joint arthritis. Both horses showed a severe pain reaction on the flexion test, with a score of 3.

After two months of daily supplementation with PEA-um, a significant improvement of lameness was observed. In particular, a lameness remission was observed for case 3, while for case 4 lameness remission was observed after three months of PEA-um treatments. These observations led to a complete return to training 6 days a week with a day of rest (5 min of walking, 15 min of trotting, and 10 min of cantering, with one jumping session per week; 400 m length with 12 obstacles, including 7 vertical obstacles and 5 large obstacles of 1.10 ± 0.10 m in height) and a return competitions once a month (class 120/130 height). To date, there have been no recurrences in lameness.

## 4. Discussion

Horse lameness is often associated with musculoskeletal inflammation, articular pain and joint disorders and is the main cause of a loss of performance in athletic horses, as well as being an important factor in the well-being and quality of life of horses [15]. Joint disease such as arthritis or injury to the tendons is often associated with pain and/or tissue inflammation. It is well-known that there is a relationship between inflammation, immune system activation, and pain sensitization across the peripheral and central nervous system [16]. Therefore, disease of the articular tissue or peripheral nerve, often due to compression and trauma, or inflammation, ischemic, and metabolic disorders, can produce a persistent inflammation and/or persistent neuropathic pain condition [17]. Additionally, navicular syndrome is one of the main causes of loss of performance in sport horses and is also an important disabling condition. Due to the presence of chronic pain in the region of the hoof capsule, it has been observed that the pathogenesis and progression of the disease includes a complex series of multi-factorial events, such as complex interactions between biomechanical stress, circulatory disorders, and above all chronic inflammation [18]. A gold standard in the management of horse lameness is to minimize the pain and inflammation, thus increasing the tissue function and slowing disease progression [19]. To obtain these results, natural nutraceutical molecules represent a family of naturally occurring lipid amides acting through the so-called autacoid local injury antagonism ALIA mechanism, and PEA is the most well-known and has been studied since 1950. PEA has emerged as a disease-modifying agent in several pathological conditions, such as inflammatory and neuropathic pain, in experimental and naturally occurring conditions in both humans and animals [20]. PEA’s mechanism of action is peculiar and involves interactions with several biological pathways, such as the endocannabinoid system, and the multitarget mechanisms of PEA exhibit a fascinating pharmacological strategy that acts according to the natural biomodulation of body responses to different stimuli and injury [21]. In particular, an important clinical study has demonstrated the effectiveness of PEA in the treatment of pain due to nerve compression, revealing its safety and efficacy compared with opioid analgesics, even in the treatment of patients not responsive to pregabalin [22,23]. Therefore, the actions of PEA on both peripheral injured tissues [24] and the pro-homeostatic central effect on pain sensitization [24] have been shown to be effective in a complex disease, such as arthritis or osteoarthritis [25,26,27]. Through this study, we have provided evidence for a prospective use of PEA in horse joint management, such as in the management of long-term articular lameness due to persistent pain. Due to the limited cases presented here as a pilot study on a very small group, future studies are needed to better understand the molecular mechanism of action of PEA in horses. Although not completely blinded to the treatment, the clinicians were unaware of the horse’s name or treatment, before examining and recording their lameness scores. This may increase the validity of the results, even if it does not completely address the reduction in the bias to a minimum and does not solve the problem of subjective variability in the evaluation of lameness and AAEP scoring. The four cases presented in this study, although differing in the etiopathology, all presented a joint disease condition associated with pain and lameness in athletic horses. The first two cases included a 7-year-old Italian gelding and a 16-year-old NRPS gelding jumping horse, respectively, with navicular syndrome conditions associated with hoof pain [4]. In particular, case 2 presented a complicated chronic navicular syndrome with distal interphalangeal joint arthrosis of the right anterior limb. In conclusion, both cases showed a significant improvement after PEA-um treatments. Both horses returned to complete training and competitions, and to date, there have been no recurrences in lameness. These observations can be partly explained by the important protective action of PEA on neuropathic pain [28]. In fact, several clinical trials in human medicine have been conducted on the use of PEA in joint diseases and neuropathic pain. PEA has been shown to be effective in the treatment of neuropathic pain in human medicine, as in nerve compression syndromes, sciatic pain, pain due to carpal tunnel syndrome, and in nerve impingement models [7,22]. In addition, the molecular mechanism of action of PEA has been investigated; in fact, the ability of PEA to reduce pro-inflammatory markers both at the peripheral and at central nervous system level has been seen, as well as its regulatory action on immune cells that play a fundamental role in inflammatory processes and therefore in algogenic sensitization [29,30].

The other two cases presented in this study had a diagnosis of distal intertarsal joint arthritis and inconsistent grade II and grade III out of V on the AAEP lameness scale. A significant improvement in horse lameness that led to them completely resuming training and competitions was observed 2 months after daily treatment with PEA-um. For these horses, to date, there have been no recurrences in lameness. These studies are consistent with the PEA effects observed in previous studies. In an experimental model of osteo-arthritis induced by iodium monoiodoacetate (MIA), treatment with PEA has been shown to produce an improvement in locomotor function as well as a protective action on cartilage, in addition to an important anti-inflammatory and pain-relieving action [25]. The protective action of PEA in joint diseases has been confirmed in human medicine as a potential successful treatment for attenuating pain and reducing other associated symptoms of knee osteoarthritis [31]. In veterinary medicine, PEA has proven effective in the treatment of atopic dermatitis in dogs and cats [8,32], and has demonstrated potential as an effective treatment for inflammatory processes and pain in veterinary medicine [6,21].

## 5. Conclusions

Our study presents four cases of athletic jumping horses with articular diseases, such as navicular syndrome and arthritis conditions associated with a disabling lameness. Although it includes a small number of cases, our study describes for the first time the efficacy of the use of PEA-um in horses. Daily oral treatment with PEA-um produced a significant improvement in all of the horses observed. These observations, in accordance with the clinical evidence for the use of PEA in both human medicine and veterinary medicine (small animals), suggest an important therapeutic role for PEA in the long-term management of lameness due to joint disorders and pain conditions in horses. The preliminary results presented suggest PEA has potential as a treatment, however a controlled experiment to test PEA in a larger cohort of horses is required to confirm these results.

## Figures and Tables

**Table 1:** Timeline of clinical outcome of horses treated with PEA-um.

Case	*Diagnosis*	Parameter	*Baseline*	*30 Days after PEA-um*	*60 Days after PEA-um*	*90 Days after PEA-um*	*120 Days after PEA-um*
(1) 7-year-old S.I. gelding jumping horse	*Navicular sindrome*	Lameness Flexion test	1 3	1 2	0 1	0 0	0 0
(2) 16-year-old NRPS gelding jumping horse	*Navicular syndrome and distal interphalangeal joint arthrosis of the right anterior limb*	Lameness Flexion test	3 3	2 3	2 2	1 1	0 0
(3) 14-year-old Holst. mare jumping horse	*Distal intertarsal joint arthritis*	Lameness Flexion test	2 *3*	1 *2*	0 *1*	0 0	0 *0*
(4) 15-year-old NRPS mare jumping horse	*Distal intertarsal joint arthritis*	Lameness Flexion test	3 3	2 *2*	1 *1*	0 *1*	0 *0*

Lameness AAEP scale of 0 to 5: 0: Lameness not perceptible under any circumstances; 1: Lameness is difficult to observe and is not consistently apparent, regardless of circumstances (e.g., under saddle, circling, inclines, hard surface, etc.); 2: Lameness is difficult to observe at a walk or when trotting in a straight line but consistently apparent under certain circumstances (e.g., weight-carrying, circling, inclines, hard surface, etc.); 3: Lameness is consistently observable at a trot under all circumstances; 4: Lameness is obvious at a walk; 5: Lameness produces minimal weight bearing in motion and/or at rest or a complete inability to move. Flexion test scale of 0 to 3: 0: no reaction/negative; 1: mild reaction; 2: moderate reaction; 3: severe reaction.
